# Multiple antibiotic resistance as a risk factor for mortality and prolonged hospital stay: A cohort study among neonatal intensive care patients with hospital-acquired infections caused by gram-negative bacteria in Vietnam

**DOI:** 10.1371/journal.pone.0215666

**Published:** 2019-05-08

**Authors:** Lynn Peters, Linus Olson, Dung T. K. Khu, Sofia Linnros, Ngai K. Le, Håkan Hanberger, Ngoc T. B. Hoang, Dien M. Tran, Mattias Larsson

**Affiliations:** 1 Global Health program, Karolinska Institutet, Stockholm, Sweden; 2 Department of Public Health Sciences, Karolinska Institutet, Stockholm, Sweden; 3 Training and Research Academic Collaboration Sweden-Vietnam, Karolinska Institutet, Stockholm, Sweden; 4 Department of Neonatology, Vietnam National Children’s Hospital, Hanoi, Vietnam; 5 Department of Microbiology, Vietnam National Children’s Hospital, Hanoi, Vietnam; 6 Department of Clinical and Experimental Medicine, Faculty of Medicine and Health Sciences, Linköping University, Linköping, Sweden; 7 Research Institute for Child Health, Hanoi, Vietnam; 8 Department of Surgery, Vietnam National Children’s Hospital, Hanoi, Vietnam; St George’s University of London, UNITED KINGDOM

## Abstract

**Background:**

Antibiotic resistance (ABR) is an increasing burden for global health. The prevalence of ABR in Southeast Asia is among the highest worldwide, especially in relation to hospital-acquired infections (HAI) in intensive care units (ICU). However, little is known about morbidity and mortality attributable to ABR in neonates.

**Aim:**

This study aimed to assess mortality and the length of hospitalization attributable to ABR in gram-negative bacteria (GNB) causing HAI in a Vietnamese neonatal ICU (NICU).

**Methods:**

We conducted a prospective cohort study (n = 296) in a NICU in Hanoi, Vietnam, from March 2016 to October 2017. Patients isolated with HAI caused by GNB were included. The exposure was resistance to multiple antibiotic classes, the two outcomes were mortality and length of hospital stay (LOS). Data were analysed using two regression models, controlling for confounders and effect modifiers such as co-morbidities, time at risk, severity of illness, sex, age, and birthweight.

**Results:**

The overall case fatality rate was 44.3% and the 30 days mortality rate after infection was 31.8%. For every additional resistance to an antibiotic class, the odds of a fatal outcome increased by 27% and LOS increased by 2.1 days. These results were statistically significant (*p* < 0.05).

**Conclusion:**

ABR was identified as a significant risk factor for adverse outcomes in neonates with HAI. These findings are generally in line with previous research in children and adults. However, heterogeneous study designs, the neglect of important confounders and varying definitions of ABR impair the validity, reliability, and comparability of results.

## Introduction

Antimicrobial resistance (AMR), in general, is the ability of a pathogen to withstand the effects of antimicrobials. Consequently, simple infections can become lethal again without effective tools to fight them. Antibiotic resistance (ABR) in bacteria is a consequence of selective antibiotic pressure in the environment, in human and veterinary medicine and food production or cross-transmission between individuals [1–4]. With a high number of antibiotics used in a limited area, healthcare settings and intensive care units (ICU) in particular are one of the main routes of emergence and transmission for resistant bacteria [3,5–10].

Hospital-acquired infections (HAI) are infections that occur during hospitalization and are often caused by gram-negative bacteria (GNB) [11,12]. As healthcare settings facilitate the spread of resistant bacteria, HAI are more likely to be caused by resistant bacteria than community-acquired infections. HAI with ABR bacteria are more difficult to treat and are associated with increased hospitalization time and mortality [9]. AMR is estimated to be responsible for 30,000 deaths per year in Europe [13] and 23,000 in the USA [2]. Emergence and spread of AMR are particularly high in low and middle-income countries (LMIC). Of all WHO regions, it is expected to be the highest in Southeast Asia [3], leading to 38,000 deaths per year in Thailand [3,14]. The emergence of multi- (MDR), extensive (XDR) and pan-drug resistant (PDR) bacteria (for definition see Magiorakos et al. [15]) is particularly worrying since clinicians are running out of treatment options and it has been estimated that AMR will cause 10 million deaths per year by 2050 if we fail to take appropriate actions now [16]. AMR and especially MDR are thus a significant burden for global health requiring immediate and coordinated action from the global community.

Neonates (babies in their first 28 days of life) and infants (first year of life) have a premature immune system and are a particularly vulnerable population for infectious diseases and hence resistant bacteria [8,11,17–19]. In LMIC, neonatal infection rates are 3 to 20 times higher than in high-income countries (HIC) and 40% of neonatal deaths are attributable to infectious diseases in general [11,20,21]. Focusing on HAI specifically, rates of HAI in neonatal intensive care units (NICU) are 9 times higher in LMIC than in HIC. They affect 20% to 50% of all NICU admissions and fatality rates range from 12% to 52% [8,11]. The most common types of HAI are pneumonia and bloodstream infections, mainly caused by GNB with increasing rates of ABR [8,11,18,22–26].

So far, little is known about mortality and morbidity attributable to ABR infections in neonates and young infants. The length of hospital stay (LOS) can serve as a measurement of morbidity and is also associated with higher costs [27–29]. Previous research indicates an increased mortality and LOS as a measurement of morbidity in neonates and infants due to ABR [24,25,29–36]. However, most studies focus on a single pathogen, type of infection or antibiotic class. Only a few studies appropriately adjust for confounders and effect modifiers, which might bias results.

The aim of this study was to assess if multiple antibiotic resistance (MAR) is a risk factor for mortality and prolonged LOS in patients from a NICU in Hanoi, Vietnam with HAI caused by GNB.

## 2. Materials and methods

### Methods

#### Setting, data collection and inclusion criteria

A total of 327 neonates and young infants from the NICU of the Vietnam National Children’s Hospital in Hanoi were included from March 2016 to October 2017 (18 months). Data were collected from patient records. Patients were considered eligible for the study if they met the following inclusion criteria: HAI (pneumonia, bloodstream infections, surgical site infections or urinary tract infections) diagnosed by the attending physician familiar with the clinical and laboratory parameters according to the ECDC criteria [37] (e.g. fever, tachycardia, metabolic acidosis, elevated CRP levels, infiltrate in case of pneumonia) caused by antibiotic-resistant bacteria and treated in isolation care. Patients that died within 24 hours after admission were excluded, as well as those cases with culture-negative HAI. Before cases were subsequently included in our cohort, the diagnosis ‘HAI’ was verified by an experienced researcher also acquainted with the ECDC criteria. Demographic data were collected from all included patients. Further analysis focused on patients with an HAI caused by *Klebsiella pneumonia*, *Pseudomonas aeruginosa*, *Acinetobacter baumanii*, *Escherichia coli* and/ or *Serratia marcescens* (n = 296) to exclude possible intrinsic resistances in gram-positive as well as in fungal species.

#### Study design and variables

The study was designed as a prospective cohort study with all-cause mortality and the LOS as the outcomes assessed in two models. The two endpoints were either ‘death’ or ‘discharged healthy’. In this setting, patients with a fatal diagnosis are frequently withdrawn from treatment, with death at home as the most common outcome. Those patients were added to the crude mortality rate with ‘death’ as outcome, the date of discharge was defined as endpoint due to loss of follow up after discharge.

The exposure of interest was the antibiotic resistance pattern of the bacteria causing the HAI. The definition of this variable differs across earlier studies. In order to provide comparability within the field, a group of international experts published definitions of MDR, XDR, and PDR for different bacterial species, based on the number of antibiotic classes a species is resistant to. Resistance was defined as non-susceptibility to at least one agent of an antibiotic class [15]. The data collected was based on the local ABR testing practice for GNB and hence did not cover all classes required to meet the definition. Therefore, it was not possible to code the exposure as a binary variable according to this classification for MDR, XDR, and PDR. To ensure some comparability nonetheless, we summed the number of antibiotic classes a species was resistant to, as did Magiorakos et al. However, we used the continuous variable for the analysis without dichotomization. The different classes (and respective agents) were: antipseudomonal cephalosporins (ceftazidime, cefepime), antipseudomonal penicillins (piperacillin/ tazobactam, ticarcillin), antipseudomonal carbapenems (imipenem, meropenem), antipseudomonal fluoroquinolones (ciprofloxacin, levofloxacin), aminoglycosides (amikacin, gentamicin, tobramycin), monobactams (aztreonam), polymyxins (colistin), folate pathway inhibitors (trimethoprim/sulfamethoxazole).

Based on previous literature [27,38–41], we added different confounders and effect modifiers as co-variates to our model:

We included the effect modifiers ‘age at admission’ and sex. Gestational age (pre-term vs. full-term) was strongly correlated with birthweight, causing multicollinearity. Therefore, we picked birthweight as a continuous variable to adjust for prematurity in our model. We included the ‘time at risk’ as a confounder, referring to the time from admission until a positive culture was obtained marking the onset of the HAI. The emergence of ABR is time-dependent and exposure to healthcare bears the risk of acquiring a resistant strain—the longer the stay, the higher the risk [40]. Consequently, a prolonged LOS can cause ABR. However, we aimed to assess LOS as a consequence of ABR and hence needed to control for the time at risk before the onset of HAI.

Secondly, we included the number of common co-morbidities to adjust for underlying diseases, namely: respiratory distress syndrome, persistent ductus arteriosus, pulmonary hypertension, hyperbilirubinemia, congenital heart disease, intraventricular hemorrhage, epilepsy, megacolon, bronchopulmonary dysplasia, necrotizing enterocolitis, hydrocephalus, and down syndrome.

Furthermore, the severity of illness must be considered. For adults, validated and updated scores including clinical and laboratory information are often applied. In neonates, several scores exist(CRIB: clinical risk index for babies; SNAP[-PE]: score for neonatal acute physiology [perinatal extension]; NMPI: neonatal mortality prognosis index; NTISS: neonatal therapeutic intervention scoring system; NBRS: neurobiological risk score);, however, every score has considerable disadvantages [42,43]: Most scores were designed in the last century and are therefore not adjusted to the medical progress made in neonatal care. Furthermore, most existing scores were designed to predict mortality in premature neonates with a range of problems, not specifically to assess the severity of an infection. This explains why certain scores (CRIB, SNAP, NMPI, SINKIN) are only validated for the first 12 or 24 hours of life, others only for neonates with very low birthweight (CRIB, CRIB II, Berlin score) and are therefore not applicable to our cohort with neonates and young infants, both premature and mature. Others (SNAP, SNAP-PE, NTISS) require a vast number of parameters which is not feasible in our setting. To control for ‘severity of illness’ nevertheless, we collected those parameters available and recommended in most scoring systems, namely birthweight, gestational age, gender and malformations, here labelled ‘co-morbidities’. As mentioned earlier, these factors were entered as confounders in the regression model, except gestational age which was later excluded because of multicollinearity. Most scores furthermore mention respiratory failure (CRIB, SNAP, SNAP-II, Berlin score, NMPI, SINKIN, NBRS, NTISS) as an important determinant of the severity of illness, others low urine output (SNAP-II, SNAPPE-II, NTISS), seizures (NBRS, SNAP, SNAP-II, NTISS) or metabolic acidosis (CRIB, CRIB II, Berlin score, NBRS, SNAP, SNAP-II, NTISS). Most of these conditions require invasive procedures such as endotracheal intubation, a urinary tract catheter or a venous catheter to administer treatment. Therefore, the sum of invasive devices (endotracheal tubes, urinary tract catheters, central and peripheral venous catheters) needed during the entire stay served as a surrogate parameter, similar as in the NTISS, and was entered as a confounder in the regression model. In ideal circumstances, an updated, reliable and validated score applicable to our cohort including feasible parameters would present the most preferable approach to adjust for ‘severity of illness’. However, including those factors widely used in existing disease severity scores for neonates (birthweight, gestational age, gender, co-morbidities) and the number of invasive devices as a proxy seems the most feasible approach to control for ‘severity of illness’ given the limitations of existing scoring systems and our setting.

Crude mortality was added as a co-variate to the model assessing LOS to avoid bias due to the phenomenon known as ‘competing risks’. In short, neonates dying during in-hospital treatment have a shorter LOS than those that survive. Assuming that ABR is associated with mortality, it would wrongly be linked to a shorter LOS if not adjusted for.

Some research indicates that polymicrobial infections (infections caused by multiple pathogens) are associated with ABR and mortality [44]. We intended to control for this possible confounder, however, it was strongly correlated with the exposure MAR and hence excluded because of multicollinearity.

#### Laboratory procedures

Laboratory information regarding culture type (tracheal aspirate or nasopharynx culture in pneumonia, blood culture in bloodstream infections, urine culture in urinary tract infections, wound culture in surgical site infections), culture date and result (isolated pathogen and resistance testing) was obtained from patients’ records. The procedures respected the guidelines of the Clinical and Laboratory Standards Institute. Antibiotic resistance was tested using the VITEK2 system (bioMérieux) and confirmed by broth microdilution, since the VITEK2 system is not reliable for all substances, e.g. colistin. Intermediate resistance was considered resistant.

#### Statistical analysis

Statistical analysis was performed using IBM SPSS version 21. Demographic characteristics, type of HAI, pathogens isolated and their ABR patterns were described with mean, median and standard deviation. The case fatality rate and the 30 days mortality rate from admission and infection were calculated. Descriptive statistics are based on the original data set. Prior to conducting multivariable analyses, missing data were handled using multiple imputation. Variables were tested for normal distribution, heavily skewed variables were log transformed to approach normality prior to the multiple imputation process and re-transformed afterwards. Multiple imputation was performed before categorizing variables to prevent loss of information. Since multiple imputation might weaken the influence of effect modifiers on the outcome, the dataset was split according to the dichotomous variable ‘gestational age’ (pre-term vs. full-term), which was assumed to be the strongest effect modifier in this study [45] and merged again after the multiple imputation process. The number of datasets generated was determined using the formula suggested by Newgard and Haukoos [46], aiming for a relative efficiency of at least 95%, which is considered a high rate. A table giving an overview of the proportion of missing data in each variable is provided in the supporting information (S1 Table).

Afterwards, two regression models were created: a logistic regression model with mortality as a binary outcome and a linear regression model with LOS as a linear outcome, both with MAR as exposure. The models were adjusted for the co-variates discussed earlier. All variables were entered simultaneously. With a sample size of n = 296 and up to eight co-variates entered in the models, the power level was sufficient to detect even small effect sizes (power level set at 80%) [47–49]. Results were considered significant with a *p*-value smaller than 0.05.

Table 1 summarizes the research problems as well as the conceptual framework and the statistical analyses used to address them.

**Table 1:** Overview of methodological approach.

1. Descriptive statistics in neonates with hospital-acquired infections (HAI) (n = 327)		
Description of study population	Demographic characteristics, type of hospital-acquired infections, pathogens isolated, antibiotic resistance patterns	
Description of outcomes	case fatality rate, 30 days mortality rate, length of hospital stay	
*- Multiple imputation -*		
2. Risk factors for adverse outcomes in neonates with HAI caused by gram-negative bacteria (n = 296)		
Outcome	Mortality	Length of hospital stay (LOS)
Exposure	Multiple antibiotic resistance (MAR) (number of resistances to different antibiotic classes)	
Regression model	logistic regression	linear regression
Co-variates (confounders and effect modifiers)	time at risk number of co-morbidities number of invasive devices sex age at admission birthweight	time at risk number of co-morbidities number of invasive devices sex age at admission birthweight crude mortality
Excluded co-variates (because of multicollinearity)	gestational age polymicrobial infection	

Since linear regression is sensitive to outliers, those outliers exceeding three standard deviations were identified (two cases in the original dataset and between no case and four cases in the imputed sets). The Cook’s distance, however, did not show a value larger than one in any of those cases (maximum of 0.054 in original data set and 0.092 in imputed data sets), therefore the cases were kept in the analysis.

#### Ethical considerations

The study was strictly observational, no interventions were performed, data was collected from patients’ records only without influencing further treatment. Identies of the children where not included in the data but a uniqe identifier fo each patient. Therefore, the caretaker’s consent was not required according to the ethical board of the Vietnam Children’s Hospital when ethical approval was given in 2015.

## 3. Results

### Descriptive statistics

#### Demographic characteristics

Out of 327 participants, 62.4% were male and 37.6% female. Almost two third were born pre-term (64.2%). The main mode of delivery was vaginal (60.9%), followed by a planned cesarean section (27.2%) and a smaller number of emergency cesarean sections (5.8%). Table 2 displays further demographical characteristics of the study population.

**Table 2:** Demographical information.

	*n*	Mean	Standard-deviation	Median	Minimum	Maximum
**Birthweight in gram**	*315*	1984.3	891	1800	600	4500
**Gestational age at birth in weeks**	*303*	34.0	5.2	34	24	40
**Age at admission in days**	*326*	8.0	9.2	4	0	44
**Weight at admission in gram**	*318*	2011.2	924.8	1815	600	4800

Total n = 327, included number of participants due to missing data given respectively

#### Hospital-acquired infection and causative pathogens

The predominant HAI were pneumonia (81.3%) and sepsis (49.2%). Sepsis referred to infections with positive blood cultures and included both primary bloodstream infections and secondary sepsis resulting from pneumonia. Other HAI were meningitis, gastroenteritis (0.9% each), surgical site infections and urinary tract infections (0.3% each). The pathogens mainly isolated from the infection site were *Acinetobacter baumanii* (27.9% of all isolates), *Klebsiella pneumoniae* (25.3%), *Pseudomonas aeruginosa* (20.5%), *Escherichia coli* (9.0%), *Serratia marcescens* (2.7%), *Staphylococcus aureus* (MRSA, 1.9%).

In total, 58.4% of HAI were caused by one pathogen, 27.5% were caused by two and 14.1% by three or more pathogens.

Since the study focuses on GNB, further calculations were based on HAI caused *by Acinetobacter baumanii*, *Klebsiella pneumonia*, *Pseudomonas aeruginosa*, *Escherichia coli* or *Serratia marcescens* as at least one of the isolates (n = 296, 90.5% of all cases).

#### Antibiotic resistance in gram-negative bacteria causing hospital-acquired infections

The proportion of ABR in the isolates ranged from 10% (colistin resistance) to over 97% (cephalosporin resistance). Table 3 shows the proportion of ABR to different antibiotic classes displayed by the main isolates.

**Table 3:** Hospital-acquired infections with, and antibiotic resistance in *Acinetobacter baumanii*, *Klebsiella pneumoniae* and *Pseudomonas aeruginosa* in relation to treatment outcomes.

	Acinetobacter baumannii			Klebsiella pneumoniae			Pseudomonas aeruginosa		
	% (n)	LOS	CFR (n)	% (n)	LOS	CFR (n)	% (n)	LOS	CFR (n)
**Hospital-acquired infections (HAI)**									
Sepsis	41.0 (66)	27.7	67.7 (44)	45.3 (73)	27.8	58.2 (39)	31.8 (50)	31.5	58.3 (28)
Pneumonia	44.0 (117)	29.3	60.2 (65)	39.8 (106)	32.3	51.0 (49)	33.8 (90)	33.2	54.4 (43)
Other HAI	37.5 (3)	39.0	33.3 (2)	37.5 (3)	38.3	50.0 (3)	16.7 (3)	44.0	40.0 (2)
**Antibiotic resistance (ABR)**									
Cephalosporins	97.2 (139)	30.0	59.7 (77)	93.1 (121)	30.8	51.9 (56)	89.4 (93)	32.6	53.7 (44)
Penicillins	92.3 (132)	31.1	60.7 (74)	90.0 (117)	31.4	51.9 (54)	91.3 (95)	33.8	48.8 (40)
Carbapenems	88.1 (126)	31.8	62.9 (73)	81.5 (106)	32.5	53.8 (50)	88.5 (92)	33.2	54.4 (43)
Fluoroquinolones	77.6 (111)	32.9	64.1 (66)	71.5 (93)	33.9	54.3 (44)	81.7 (85)	34.9	52.1 (38)
Aminoglycosides	92.3 (132)	31.2	61.5 (65)	93.1 (121)	30.4	52.8 (57)	89.4 (93)	33.0	53.1 (43)
Monobactams	25.9 (37)	33.1	61.8 (21)	58.5 (76)	32.5	56.7 (38)	39.4 (41)	34.9	52.8 (19)
Colistin	10.5 (15)	31.2	64.3 (9)	16.9 (22)	39.2	61.1 (11)	17.3 (18)	39.5	53.3 (8)
Trimethoprim/ Sulfamethoxazole	69.2 (99)	34.4	61.1 (55)	73.1 (95)	33.5	52.4 (44)	90.4 (94)	34.1	48.1 (39)

LOS = length of stay in days, here after infection; CFR = case fatality rate in percentage and in absolute numbers (n)

The first section displays the proportion of sepsis, pneumonia and other HAI caused by *Acinetobacter baumanii*, *Klebsiella pneumoniae* and *Pseudomonas aeruginosa* respectively. The sum exceeds 100% since pneumonia and sepsis were often concurrent conditions and hospital-acquired infections (HAI) were frequently caused by more than one pathogen. The second section describes the proportion of antibiotic resistance (ABR) to the respective antibiotic class in the isolated bacteria. HAI and ABR are additionally displayed by treatment outcomes—the mean length of hospital stay (LOS) after infection and the case fatality rate (CFR)—for each of the bacterial species separately.

**Fig 1:**
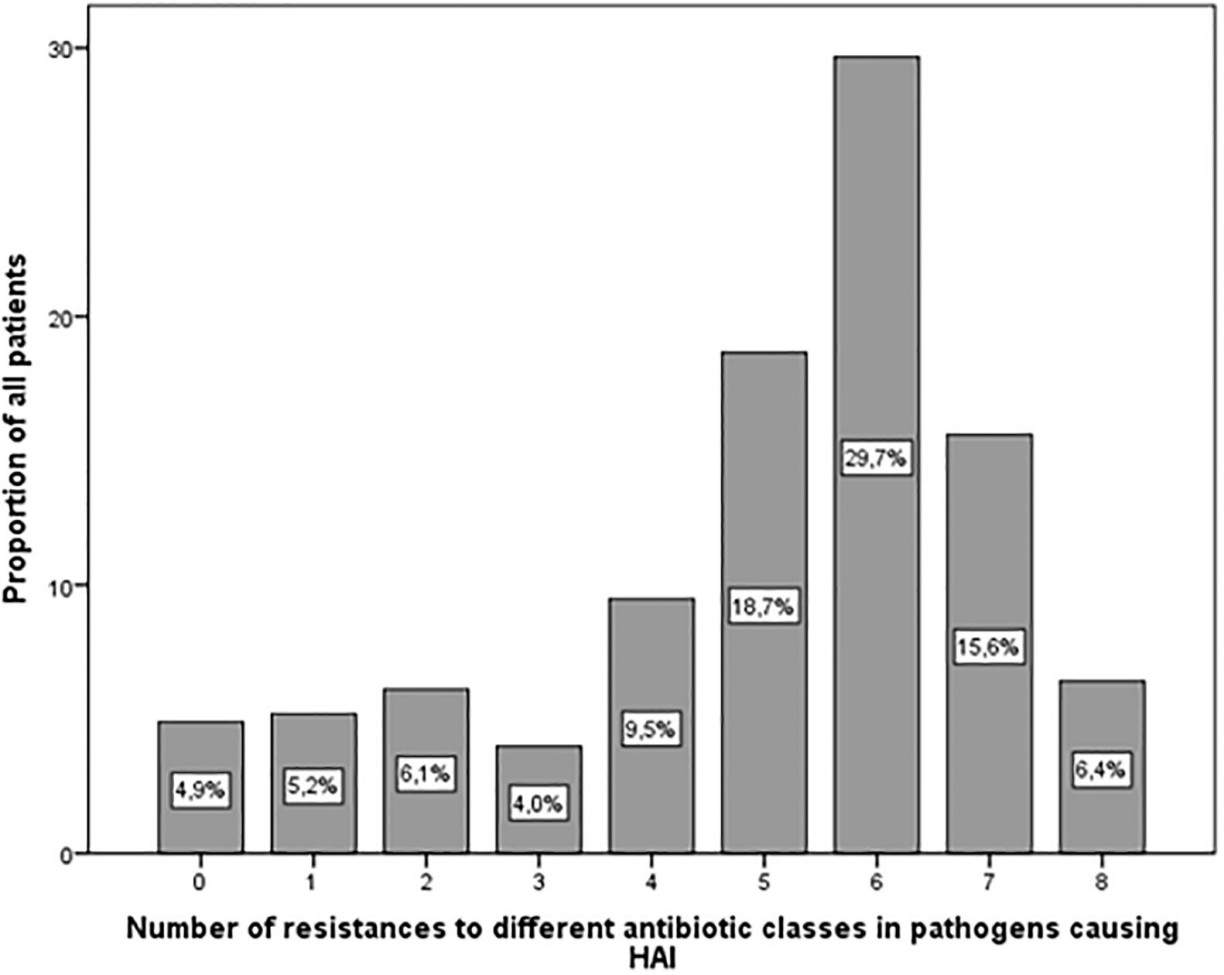
Multiple antibiotic resistance (MAR). Proportion of patients with hospital-acquired infections caused by gram-negative bacteria (GNB) with antibiotic resistance (ABR) to between 0 and 8 antibiotic classes. These classes included antipseudomonal cephalosporins, penicillins, carbapenems, fluoroquinolones, aminoglycosides, monobactams, polymyxins, folate pathway inhibitors.

This study focuses on the effect of MAR on treatment outcome. Fig 1 displays MAR, i.e. the number of different antibiotic classes isolated GNB were resistant to. Most patients (64%) had HAI caused by GNB with ABR to between five and seven antibiotic classes.

#### Descriptive statistics of outcomes

Mean total LOS in the initial study population (n = 327) was 38.7 days (SD 26.0 days) with a median of 30 days. Of all patients, 46.2% were discharged in good health while 44.3% died or were withdrawn with a fatal diagnosis (case fatality rate). Of those that died, 57.0% died within 30 days after admission and 71.7% within 30 days after infection. For the initial study population (n = 327), the 30 days mortality rate after admission was hence 25.3% and after infection 31.8%. Table 3 summarizes the outcomes in relation to the HAI and the GNB isolated.

### Multivariable analysis

#### All-cause mortality

A logistic regression model assessed the effect of MAR in GNB causing HAI in neonates on the likelihood to die while adjusting for possible confounders. The model χ^2^ (df = 7) = 21.877, *p* = 0.003, Nagelkerke’s R^2^ = 11.8% classified 62.7% of cases correctly. With every increase in the score of MAR, i.e. for every additional resistance to an antibiotic class, the odds of a fatal outcome increased by 27% (c.f. Table 4). This result was statistically significant (*p* = 0.007).

**Table 4:** Logistic regression model with all-cause mortality as outcome.

Variable	Odds ratio (OR)	95% Confidence interval	*p*-value
**Exposure:**			
Multiple antibiotic resistance (MAR) score	1.269	1.067–1.508	*0*.*007*
**Co-variates:**			
Time at risk	1.000	1.000	*0*.*579*
Number of co-morbidities	0.952	0.678–1.336	*0*.*775*
Number of medical devices	1.492	1.188–1.874	*0*.*001*
Birthweight	1.000	1.000	*0*.*618*
Sex*	0.874	0.494–1.546	*0*.*643*
Age at admission	1.007	0.979–1.036	*0*.*632*

*Sex coded as 0 = male, 1 = female; time at risk as baseline; OR > 1: risk factor, OR < 1 protective factor

Adjusting for different confounders, here listed as co-variates, multiple antibiotic resistance (MAR) was a significant risk factor for mortality, increasing the odds by 27%.

#### Length of hospital stay

To assess the effects of MAR on LOS, a multiple linear regression was conducted. A significant model *F* = 19.884 (df = 8; *p* < 0.001, standard error = 21.272) explained 41.6% (*R*^*2*^) of the outcome’s variance, which is a moderate proportion. The regression coefficients are listed in Table 5.

**Table 5:** Multiple linear regression model for ‘length of hospital stay’ (LOS) in days as outcome.

Variable	*B*	95% Confidence interval of *B*	Standard error	*p*-value
**Exposure:**				
Multiple antibiotic resistance (MAR) score	2.066	0.209–3.923	0.947	*0*.*029*
**Co-variates:**				
Time at risk in days	-1.971E-5	0.000–0.000	0.000	*0*.*913*
Number of co-morbidities	3.078	-0.791–6.948	1.973	*0*.*119*
Number of invasive devices	0.528	-2.043–3.948	1.311	*0*.*687*
Sex*	-1.654	-7.791–4.483	3.131	*0*.*597*
Age at admission in days	-0.285	-0.611–0.42	0.166	*0*.*087*
Birthweight in gram	-0.010	-0.14–-0.007	0.002	*< 0*.*001*
Crude mortality	-6.064	-12.184–0.056	3.122	*0*.*052*
	Intercept: 48.219 (95% CI: 32.247–64.191)			

*Sex coded as 0 = male, 1 = female; *B* = unstandardized regression coefficient; 95% CI *B* = 95% confidence interval of *B*

Adjusting for important confounders, here listed as co-variates, the length of stay (LOS) in the hospital increased by 2.1 days with every increase in MAR score.

Entered in the regression equation, LOS significantly increased by 2.1 days for every additional resistance to an antibiotic class. Furthermore, a lower birthweight was associated with an increased LOS, however with a smaller effect size compared to the MAR score. Mortality was almost significantly (*p* = 0.052) correlated with LOS. The two significant variables account for 11.6% (birthweight) and 2.0% (MAR score) of the outcome’s variance and therefore influence LOS to a larger extent than other independent variables.

## Discussion

In a prospective cohort study, we assessed the effect of multiple antibiotic resistance (MAR) in gram-negative bacteria (GNB) causing hospital-acquired infections (HAI) in Vietnamese neonates and young infants on mortality and the length of hospital stay (LOS). The isolates showed a high level of resistance against multiple antibiotic classes. Higher rates of antibiotic resistance were significantly associated with higher mortality and a longer hospital stay when confounding factors were held constant.

### Hospital-acquired infections and antibiotic resistance patterns and their implications for global health

The most frequent HAI were pneumonia and sepsis. Other HAI were meningitis, gastroenteritis, surgical site infection, and urinary tract infection. These findings were similar to previous research in the same [18] or different settings [25,26,50].

Eighty-five percent of all isolates causing HAI were GNB. The bacteria mainly isolated were *Acinetobacter baumanii* (28%), *Klebsiella pneumoniae* (25%) and *Pseudomonas aeruginosa* (21%), followed by *Escherichia coli* (9%) and *Serratia marcescens* (3%). Most studies investigating sepsis [23,24,51–53], pneumonia [54] or HAI in general [11,12,18,25,26] found GNB as the main causative pathogens for neonatal HAI, fewer name gram-positive bacteria as the main agent [45,55,56]. The exact bacteriological profile of HAI depends on the setting and the type of HAI investigated. Nevertheless, *Acinetobacter baumanii*, *Klebsiella pneumoniae*, *Pseudomonas aeruginosa*, and *Escherichia coli* were predominantly isolated in prior research, similarly to our study [18,24–26,51–54].

During the last decade, research increasingly focuses on multidrug resistance (MDR), extensive drug resistance (XDR) and pan-drug resistance (PDR) [15,57]. However, the use of these terms varies widely between studies, especially before Magiorakos et al. [15] offered a comprehensive definition of those classifications for common strains (Magiorakos et al. [15]: ‘MDR was defined as acquired non-susceptibility to at least one agent in three or more antimicrobial categories, XDR was defined as non-susceptibility to at least one agent in all but two or fewer antimicrobial categories (i.e. bacterial isolates remain susceptible to only one or two categories) and PDR was defined as non-susceptibility to all agents in all antimicrobial categories.’). Previous studies investigating neonatal HAI found MDR in 11% [58], 35%-55% [24,25,32] and in 80% of all isolates [33,59]. The global reports on infections due to XDR and even PDR GNB are increasing and a cause for concern [60–62]. Teerawattanapong et al. [57] detected a particularly high burden of *Acinetobacter spp*. with MDR, XDR or PDR in Southeast Asia compared to other regions. The exact definition suggested by Magiorakos et al. [15] could not be applied to the study at hand since bacteria were not tested for all required antibiotic classes, e.g. tetracyclines since they are contraindicated in neonates. Nevertheless, 70% to over 90% of isolates were resistant to antipseudomonal penicillins, cephalosporins, carbapenems, fluoroquinolones, aminoglycosides, and trimethoprim/ sulfamethoxazole, suggesting a high rate of MDR and a considerable rate of XDR. However, these figures do not represent the prevalence of ABR in the area, which is presumably lower. Since the study was conducted in a tertiary hospital with many patients referred to due to infections, the numbers are overestimating the true prevalence of ABR. Le et al. [18] showed in an ICU point prevalence survey of HAI in five Vietnamese pediatric hospitals including a total of 1363 cases a carbapenem resistance between 55% in *Klebsiella pneumoniae* to 71% in *Acinetobacter baumanii* and a prevalence of cephalosporine resistance of 90% in *Klebsiella pneumoniae*.

The case fatality rate was 44.3% and similar to previous research indicating an overall neonatal fatality of 38 to 47% [19,53,63]. Of those that died, 71.7% died within 30 days after infection while Saleem et al. [19] report a similar proportion (70%) after only four days.

Comparing mortality as a consequence of ABR across the literature is challenging because of vast methodological differences. Risk factor and outcome studies are frequently combined, although not recommended [64] and study designs differ greatly. Unfortunately, not all investigators conduct multivariable analysis to adjust for confounders or effect modifiers. Although some papers [27,38–40] emphasize the need to control for co-morbidities, the severity of illness and the time spent in a health facility before infection (time at risk), this is not yet common practice, possibly leading to biased results. The co-variates included vary across the field, sometimes exceeding the capacity of the sample size, decreasing the power.

### Antibiotic resistance as a risk factor for mortality

In our study, multiple antibiotic resistance (MAR) in GNB remained a significant risk factor for mortality in neonates with HAI after adjusting for potential confounders. This association might be attributable to changes in the bacteria to withstand the host’s immune defense, however, several studies report a reduced fitness in resistant strains compared to susceptible strains [65–67]. Consistent with prior research, we assume that delayed appropriate treatment and the absence of effective drugs in cases resistant to all tested substances contribute to the increased mortality in our cohort since empiric treatment might fail due to ABR [36,68–71]. Data about the exact dates and dosage of antibiotic therapy were not available to us, hence we lack strong evidence to support our hypothesis. However, the authors observed that although local recommendations for antibiotic treatment consider the prevalence of ABR in this setting, first line antibiotics were often ineffective in patients with HAI caused by MDR pathogens. Consequently, appropriate treatment is delayed by the time clinicians receive microbiological test results including sensibility testing. In those cases where all antibiotics were tested resistant (6.4%) and empirical therapy was therefore certainly inadequate, mortality was highest. In these patients, a combination of different substances was administered, hoping for a residual antibiotic effect in vivo.

So far, not many studies investigated mortality due to ABR in neonates. Focus often lies upon a single type of infection, bacteria or antibiotic class, studies including multiple antibiotic classes are rare.

In general, our study confirms previous findings. While only a few studies did not identify ABR as a risk factor for mortality in neonates [72], the majority have found significant associations. For instance, mortality was reported to be higher in patients infected by ESBL-producing GNB [29,31] or by GNB with carbapenem resistance [30], increasing the odds of dying 3- to 4-fold.

Most research focusing on resistance to multiple antibiotic classes indicates an association with mortality. Al Jarousha et al. [32] found a mortality rate of 37.5% in NICU patients infected with MDR *Acinetobacter spp*. compared to only 12% in controls. However, controls were uninfected, so the result might be more attributable to the infection itself than to ABR. Folgori et al. [25] found a 30 days mortality rate of 19% in patients with HAI caused by MDR strains compared to 13% in susceptible strains (*p* = 0.06) in neonates and children. Others [24] reported similar results with a fatality rate of 16% in patients with sepsis caused by MDR bacteria compared to 12% in those with susceptible strains and 8% in controls with culture negative sepsis.

However, only a few studies adjust for confounders, which is essential if ABR is claimed to be a risk factor for mortality. In two elaborate studies which included two control groups each and adjusted for confounders, Thatrimontrichai *et al*. [33,34] reported carbapenem resistance to be a significant risk factor for mortality in patients with infections caused by *Acinetobacter spp*. The mortality was significantly higher in bloodstream infections caused by resistant strains compared to those caused by susceptible strains (OR = 5). These results are similar to ours, as we identified MAR as a risk factor for mortality with an OR of 1.3, however assessing a broader spectrum of bacteria, infections, and antibiotic classes.

### Prolonged length of hospital stay attributable to antibiotic resistance

Studies assessing LOS in neonates are scarce and varying with respect to the type of infection, pathogen, and antibiotic resistance pattern. Abdel-Hady et al. [31] and Sehgal et al. [29] found that the hospital stay was prolonged in patients infected with ESBL-producing GNB. In a study conducted by Thatrimontrichai et al. [34], hospital stay was longer in patients with pneumonia caused by carbapenem-resistant *Acinetobacter spp*., however, the result was not significant. In children admitted to an ICU, infections with a resistant strain seem to prolong the hospital stay compared to infections caused by a susceptible strain in some studies [73], others found no significant difference [74].

Focusing on MDR, despite the varying definition of this variable, Mauldin et al. [75] assessed the LOS in patients from all age groups admitted to the general ward or ICU with HAI. They and concluded that LOS was prolonged by 24% in patients infected with MDR strains. Other studies including adults [9,71,76] also indicate a longer hospital stay if an infection is caused by resistant compared to susceptible bacteria.

However, the vast majority of those studies did not control for confounders, especially for the ‘time at risk’. Exposure to healthcare is a risk factor to acquire colonization or infection with ABR bacteria due to cross-transmission or selective pressure on the microbiome during antibiotic treatment [7,34,40]. Reversely, an infection with a resistant strain can prolong the hospital stay since it might be more difficult to treat [7,31,77]. Thus, ABR can present the cause or the consequence of a prolonged hospital stay. To ensure that only LOS attributable to ABR is assessed, ‘time at risk’, the time before infection occurs, should be entered as a confounder in the multivariate analysis [27,41]. One of the few studies controlling for ‘time at risk’ and co-morbidities found increased mortality (OR 4.4) and a longer hospital stay (hazard ratio 2) in patients with MDR *Pseudomonas spp*. infection compared to susceptible controls [78]. Lower birthweight was significantly associated with increased LOS and should therefore always be included as a confounder. Mortality was an almost significant co-variate in our regression model, demonstrating the importance to control for this confounder when assessing LOS. Our study, likewise adjusting for possible confounders, found an increase of LOS by 2.1 days for every additional resistance to an antibiotic class.

### Strengths

A strength of this study is the considerable sample size and adjustment for important confounders such as co-morbidities and ‘time at risk’ as well as for possible effect modifiers. Most studies identified birthweight or gestational age as a significant effect modifier when generally assessing mortality in neonates. Especially preterm neonates are at a higher risk for adverse outcomes with a compromised immune system and other underlying diseases [19,53]. For future studies investigating ABR-related mortality in neonates, we hence suggest including birthweight, gestational age, age at admission and sex as possible effect modifiers in the multivariate analysis.

### Limitations

Obtaining tracheal aspirate and nasopharyngeal cultures always entails a potential risk of contamination with bacterial species other than the one or those causing pneumonia. The gold standard in adults however, the bronchoalveolar lavage, can be harmful in severely ill neonates. Since the bacterial species included in this study (*Klebsiella pneumonia*, *Pseudomonas aeruginosa*, *Acinetobacter baumanii*, *Escherichia coli* and/ or *Serratia marcescens*) are not part of the typical nasopharyngeal flora, the attending physicians in the vast majority of cases treated the patients based on the assumption that the pathogens isolated using tracheal aspirates and nasopharyngeal cultures are de facto causing the HAI. Furthermore, many neonates with pneumonia developed a secondary sepsis that allowed us to confirm the causative pathogen in blood cultures.

Since we lacked the exact dates and dosages of drug administration, we cannot ultimately prove if inappropriate empirical treatment is the underlying reason for the adverse outcomes observed.

To adjust for ‘severity of illness’, the number of invasive devices was used as a surrogate parameter as well as other important factors recommended in existing scoring systems. This approach is likely to minimize the influence of ‘severity of illness’ on the outcome, however, it is no validated method and we cannot exclude residual confounding. As already discussed earlier, a more favorable approach to control for this confounder would be a validated score including clinical and feasible laboratory parameters. This score should preferably be assessed before and with the onset of an HAI to adjust for the severity of the underlying disease as well as for the severity of the HAI itself (the mortality in sepsis is presumably higher than that of a simple urinary tract infection, irrespective of ABR). Another possibility might be to adjust for the type of HAI, however, this would have reduced our sample size and with it the statistical power.

Our variable ‘time at risk’ which adjusts for previous exposure to healthcare only accounts for the time since admission to the hospital, the time of prior hospital stays in case of referral was not considered.

Because of missing information, we were unable to compare MDR and non-MDR bacteria according to the definition by Magiorakos et al. [15] and introduced the variable MAR instead. Nevertheless, we first tried the analysis with a binary variable similar to this definition. Yet no significant results were obtained. We conclude that dichotomizing a continuous variable, such as the number of resistances to different antibiotic classes, leads to loss of information and can reduce the discriminatory power. This might be a reason why several studies were unable to detect a difference between infections caused by MDR and non-MDR bacteria. Using a continuous variable as we did might, therefore, present an alternative approach.

## Conclusion

Antibiotic resistance in gram-negative bacteria, here measured as resistance to multiple antibiotic classes, is a significant risk factor for mortality and prolonged hospital stay in patients admitted to a neonatal intensive care unit with hospital-acquired infections in the Vietnamese context. The adjustment for confounders such as co-morbidities, severity of illness and time at risk is essential to prevent biased results, yet not common practice in the field.

In line with previous research, this study emphasizes that antibiotic resistance is an increasing burden for global health, especially for vulnerable patients such as neonatal intensive care patients in resource-constraint settings. Tackling antibiotic resistance must become a priority for the global community to reduce mortality, morbidity and healthcare expenses.

## Supporting information

S1 Table Proportion of missing data in the variables of interest. The table shows the percentage of missing data in each of the variables of interest across the complete dataset (n = 327).
(DOCX)

S1 Data The primary data our statistical analysis is based on. (SAV)
