# Supplementary material for: Multiple antibiotic resistance as a risk factor for mortality and prolonged hospital stay: A cohort study among neonatal intensive care patients with hospital-acquired infections caused by gram-negative bacteria in Vietnam
Source: PLoS One. 2019 May 8;14(5):e0215666. doi: 10.1371/journal.pone.0215666 (PMC6505890; doi:10.1371/journal.pone.0215666)
Supplement: S1 Table — The table shows the percentage of missing data in each of the variables of interest across the complete dataset (n = 327). (DOCX) [file pone.0215666.s001.docx]

**S1 Table. Proportion of missing data in the variables of interest**. The table shows the percentage of missing data in each of the variables of interest across the complete dataset (n =327).

| **Variable** | **Missing data (%)** |
| --- | --- |
| **Descriptives** | |
| Type of HAI | 8.6% |
| Pathogen isolated | 0.0% |
| **Outcome** | |
| Mortality | 9.5% |
| Length of stay in days | 10.7% |
| **Exposure** | |
| Antibiotic resistance to: | |
| Cephalosporines (3^rd^ & 4^th^ generation) | 3.6% |
| Antipseudomonal penicillins | 8.3% |
| Carbapenems | 1.4% |
| Aminoglycosides | 1.4% |
| Fluoroquinolones | 2.4% |
| Monobactams | 1.5% |
| Trimethoprim/sulfamethoxazole | 0.6% |
| Colistin | 46.2% |
| **Demographic data, confounders, effect modifiers** | |
| Sex | 0.0% |
| Mode of delivery | 6.1% |
| Gestational age at birth in weeks | 7.3% |
| Birthweight in gram | 3.1% |
| Weight at admission in gram | 2.8% |
| Age at admission to the hospital | 0.3% |
| Number of co-morbidities | 6.1% |
| Invasive medical devices (surrogate parameter for severity of illness) | |
| Urinary tract catheter | 60.6% |
| Central vascular catheter | 32.7% |
| Peripheral vascular catheter | 27.5% |
| Endotracheal tube | 15.9% |
